# Untangling the fusion of spatial omics and mechanobiology

**DOI:** 10.1088/2516-1091/ae16f2

**Published:** 2025-12-12

**Authors:** Samuel Dembowitz, Felix G Rivera Moctezuma, Nicholas Zhang, Abhijeet Venkataraman, Ahmet F Coskun

**Affiliations:** 1 Wallace H. Coulter Department of Biomedical Engineering, Georgia Institute of Technology and Emory University, Atlanta, GA, United States of America; 2 Parker H. Petit Institute for Bioengineering and Bioscience, Georgia Institute of Technology, Atlanta, GA, United States of America; 3 Interdisciplinary Bioengineering Graduate Program, Georgia Institute of Technology, Atlanta, GA, United States of America; 4 Woodruff School of Mechanical Engineering, Georgia Institute of Technology, Atlanta, GA, United States of America

**Keywords:** spatial, omics, proteomics, transcriptomics, mechanotransduction

## Abstract

Cellular biophysical properties are increasingly linked to disease development, including muscular dystrophy, cancer, glaucoma, and other conditions. Transcription profiles of various types have been utilized to elucidate the relationship between genes and their regulatory functions. While spatial transcriptomics creates high-resolution maps of gene regulation in tissues, it does not capture the mechanically coordinated responses of cells based on their transcriptional profiles and cell locations. Mechanobiology, on the other hand, studies how cells perceive and respond to forces but lacks genomic information. In this paper, we explore the emergence of an integrative platform called spatial mechano-transcriptomics. This method combines spatial transcriptomic and mechanical data from the same cells within a timeframe suitable for diagnostic procedures. Spatial mechano-transcriptomics examines the relationship between physical properties, including cell membrane stiffness, and differences in the cell’s transcription profile, which could be used to predict disease states. Integrating spatial and mechanical observations has the potential to revolutionize precision diagnostics and lead to the development of new therapeutics, resulting in significant advances in biomedical research.

**List of abbreviations**
RNA: Ribonucleic Acid
US: United States
ECM: Extracellular matrix
ERK: Extracellular signal-regulated kinase
LCM: Laser capture microdissection
IMS: Image mass spectrometry
mRNA: Messenger ribonucleic acid
MS: Mass spectroscopy
DNA: Deoxyribonucleic acid
GFP: Green fluorescent protein
PLA: Proximity ligation assay
RCA: Rolling circle amplification
PCR: Polymerase chain reaction
MSI: Mass spectroscopy imaging
DESI: Desorption electrospray ionization
SIMS: Secondary ion mass spectroscopy
MALDI: Matrix-assisted laser desorption ionization
SCS: Selective cell sampling
2D: Two dimensional
scSpaMet: Single cell spatially resolved metabolomics
TOF: Time of flight
IMC: Imaging mass cytometry
3D: Three dimensional
AFM: Atomic force microscopy
RT-qPCR: Real time quantitative polymerase chain reaction
TFM: Traction force microscopy
PEG: Polyethylene glycol
MEMS: Microelectromechanical systems
FRET: Förster resonance energy transfer
MII: Mechanical imaging interferometry
Yap: Yes-associated protein
PGE: Prostaglandin E
IGF-BP: Insulin-like growth factor-binding protein
Net/NTN: Netrin
LAMA: Laminin
FN: Fibronectin
KO: Knockout
WT: Wild type
BM: Basement membrane
FSS: Fluid shear stress
VMSI: Variational method of stress inference
MAPK: Mitogen-activated protein kinase
GTP: Guanosine triphosphate
FISH: Fluorescent *in situ* hybridization
SM: Small molecule
Seq: Sequencing
FISSEQ: Fluorescent *in situ* sequencing

## 1. Introduction

**Figure 1.**
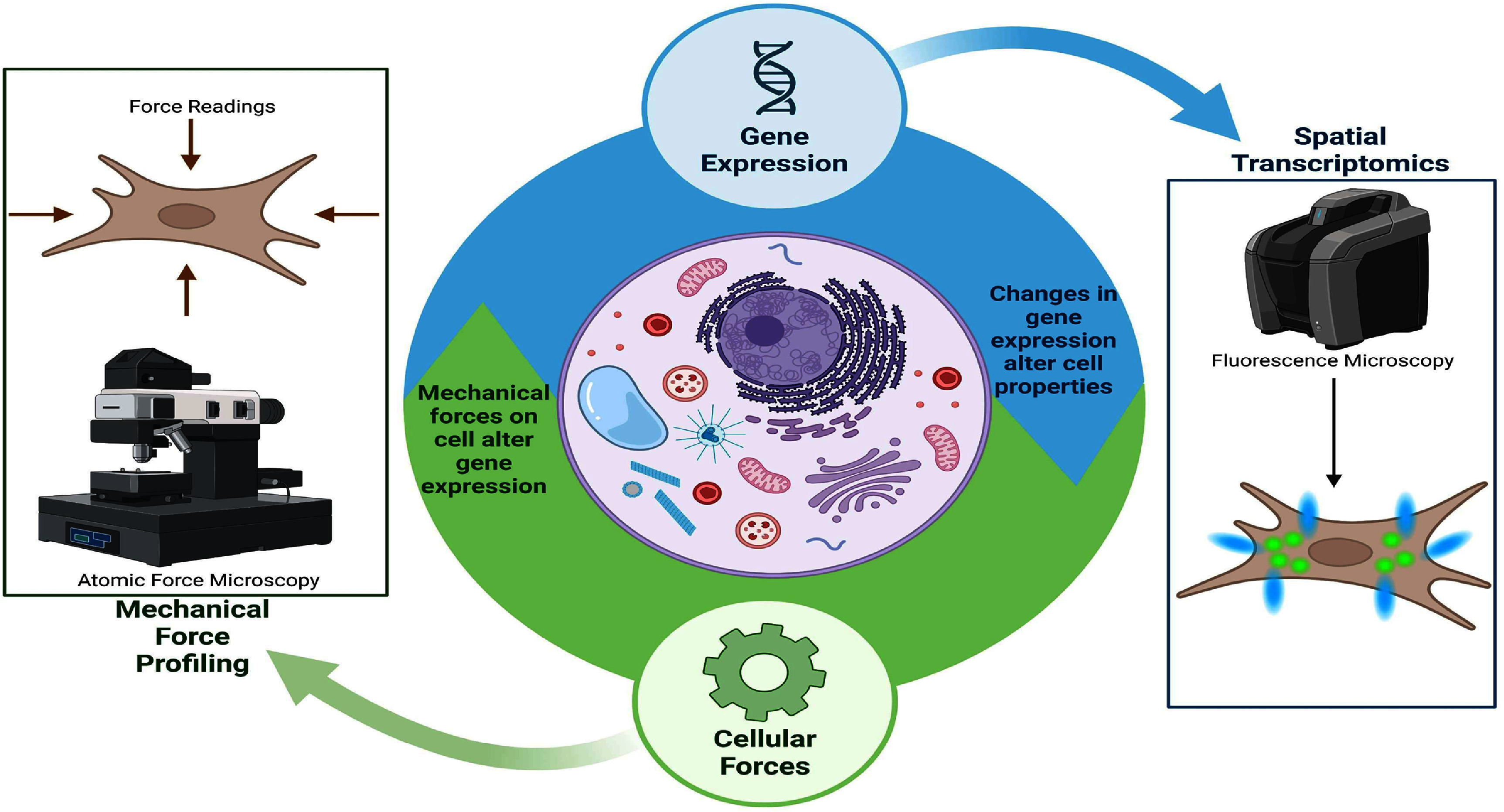
The connection between mechanical forces acting on cells and changes in gene expression is significant. Cells respond to environmental cues by adjusting their gene expression, which can be visualized with spatial transcriptomics, as depicted in the right box. The primary method for this is fluorescence microscopy, where fluorescent markers activate upon binding to specific cellular molecules. Since altered gene expression affects cell behavior, it changes the mechanical forces they exert. Monitoring these forces is challenging, but, as shown in the left box, AFM is a common technique. AFM uses tiny probes to measure cell membrane stiffness and estimate applied forces. These force changes further influence gene expression, creating a feedback loop. Created in https://BioRender.com.

With the increasing accessibility of organism-wide genetic profiling, personalized therapies have rapidly advanced. Beyond genome sequencing, spatial transcriptomics now enables high-resolution mapping of gene expression within cells and tissues, yielding rich insights into when and where specific genes are active within a cell’s genome [1]. These biotechniques fueled questions about the transcriptional changes and their influences on the cell’s behavior. In the meantime, mechanobiology has studied the role of physical forces on cell responses. However, current approaches typically perform decoupled analysis of transcriptional or mechanical aspects, hindering the interplay between the two (figure 1). Integrating these fields could provide a comprehensive view of molecular programs and physical decision-making in health and disease.

The understanding of how several diseases progress is due to advancements in both fields of medicine and research. Throughout the body, mutations related to the mechanical properties of cells have the potential to develop into diseases. The most common example of this is cancer, with an estimated prevalence of 21.6 million people in the US [2]. Modifications to cell membranes, stiffer ECMs, and other mutations have been linked to changes in the likelihood of metastasis. Another example is arteriosclerosis, which involves the stiffening of arterial fibroblasts [3]. Fibroblasts exhibit varying forces to FSS [4]. Behavioral changes, such as diet and exercise, can alter gene expression in fibroblasts, influencing their resistance to shear stress. Other mechanical changes in cells have affected over 250 000 people in the US through the progression of muscular dystrophy [5]. Despite multiple diseases that can cause muscular dystrophy, biological signs are measured before other symptoms appear. In skeletal muscle cells, genes are linked to changes in the ECM-cell connection. A weakening of this connection leads to a decrease in ERK 1 and 2 proteins, resulting in the degeneration known as muscular dystrophy. Many diseases can be traced back to variations in the mechanical behavior of cells, underscoring the importance of a better understanding (table 1).

**Table 1.** Selection of a few diseases that are associated with mechanobiology changes. Prevalence ranges are determined by current research about the number of patients and the growth of the condition.

Disease	US prevalence	Mechanical changes	References
Cancer	18–20 million	Several modifications to many cell types, including stiffer cell membranes, stiffer ECM, Rho family of GTPases increase, etc.	[2, 6]
Arteriosclerosis	19–20 million	Fibroblasts with reduced resistance to everyday fluid shear stress	[3,7]
Deafness	30–48 million	Mutated genes reduce the number of tip proteins, reducing cell surface tension	[8,9]
Glaucoma	1–5 million	Higher intra-ocular pressure triggers fibroblast remodeling by activating tenascin C	[9, 10]
Polycystic kidney disease	250 000–400 000	Mutation in transient receptor potential channel family protein polycystin-2 prevents transduction from occurring, causing the growth of new cells	[9, 11]
Muscular dystrophy	200 000–300 000	Mutated gene increases ECM-cell stretch, resulting in higher ERK 1 and 2 transcription	[5, 9]

**Figure 2.**
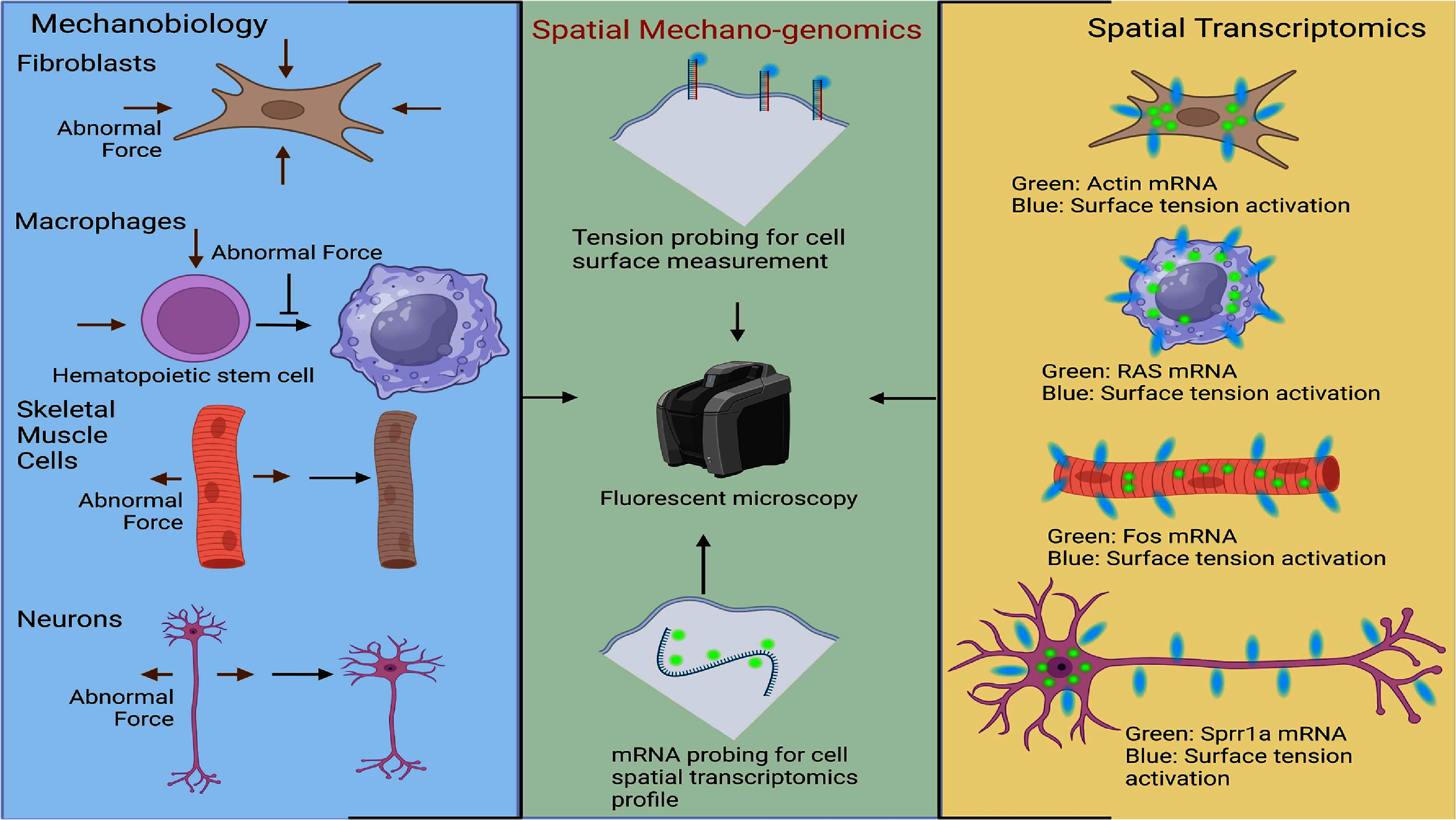
Comparison between spatial transcriptomics and mechanobiology across multiple cell types. The left panel illustrates previous findings on how fibroblasts, macrophages, skeletal muscle cells, and neurons respond to various mechanical forces. Mechanical stimuli affect fibroblast growth, macrophage differentiation, and MAPK pathway regulation in skeletal muscle cells. Neurons show alterations in growth and regeneration when subjected to different mechanical stresses. The right panel displays results from various fluorescent markers on the cell surface and within the cell. Green indicates intracellular mRNA, while blue highlights surface receptor tension probes. These markers differ depending on cell type and may also be influenced by mechanical force activation. The central part of the figure hints at a fusion of mechanobiology and spatial transcriptomics, termed spatial mechano-genomics. Combining these approaches enables visualization of gene transcription linked to mechanical changes in cells. Created with https://BioRender.com.

Currently, reports tend to focus either on the mechanical forces of cells or on spatial transcriptomics. This separation causes common limitations such as delays over time and potential misinterpretations, which can impact how results are correlated. When examining the spatial transcriptomics of a diseased cell, the cell’s physical properties cannot be observed. Additionally, the underlying genetic changes are not visible when only looking at changes in the cell’s behavior. This review highlights the gap between spatial transcriptomics and mechanobiology and explores an opportunity to develop a new method (figure 2) that provides unprecedented insights into the mechanical behavior and transcriptional profile of cells.

## 2. Spatial transcriptomics methods map the genetic profile of cells

The analysis of cellular gene expression has greatly enhanced our understanding of cellular heterogeneity and the roles of various cell types. The main challenge with most cellular gene expression studies is the need to remove the cell from its surrounding tissue while maintaining its integrity [12]. Even when this is successful, the cell can lose some of its properties. Spatial transcriptomics, on the other hand, involves analyzing gene expression in cells without removing them from their anchored tissues. This approach enables the study of more complex tissues, such as those in the nervous system. By examining cells within their native tissue context, researchers can expand and validate findings from previous experiments.

For example, examining the spatial omics of the ECM more closely reveals its relationship to heterogeneity. There have been several discussions about the benefits and drawbacks of several spatial transcriptomic techniques (table 2) [1]. Current methods for classifying the proteins and structures of the ECM indicate that no single method can collect all the necessary information. LCM and ECM-IMS are potentially effective techniques for analyzing protein patterns in ECM [1]. Additionally, understanding the architectural organization and mechanical properties of the ECM is equally important, but it requires techniques like AFM, which are not directly related to spatial omics [1].

**Table 2.** Overview of spatialomics methods currently used in laboratory settings [1, 13].

	Image-based spatial transcriptomics	Sequencing-based spatial transcriptomics	Spatial proteomics
Methods	smFISH, MER FISH, seq FISH, *In-situ* seq	*In-situ* seq, slide seq, FISSEQ, DBiT-seq	Fluorescent proteins, Halo tags, enzyme binding, biarsenical dyes, multiplexed, sequential, and cyclic imaging
Modality	Fluorescent labeling and tracking of genetic information at subcellular resolution	Amplify gene expression profile and sequence a few cell resolution	Tag proteins for fluorescent imaging, DNA barcodes
Application to mechanobiology	Illustrate changes in genetic transcription profile through fluorescent localization of specific sequences within the cell. Several markers can identify changes in communications and mechanical connections between cells.	Illustrate changes in gene transcription through barcoded DNA images that localize specific sequences and show their concentrations in one concise image. These images can also compare cell-type responses to mechanical stimuli.	Illustrate downstream effects of mechanical stimuli via fluorescence labeling of multiplexed proteins on the cell surface and inside the cell. These proteins also show changes in cell communication due to mechanical changes.

Although the technology is only about a decade old, the field is rapidly advancing with more efficient techniques and increased computing power. Several methods are employed in spatial transcriptomics, which fall into different categories [12]. One involves hybridizing fluorescent markers to mRNA in the cells for analysis via microscopy [12]. Another approach focuses on sequencing the mRNA from the cells to create profiles of their gene expression. Many have utilized spatial transcriptomics to elucidate the differences between healthy and diseased cells in various regions of the body [12]. Additionally, detailed models of diseases like breast cancer have been developed by studying tissue architecture and cell communication. The amount of actionable information the images can provide about cell or tissue properties is limited.

### 2.1. Spatial proteomics

Spatial proteomics, the study of protein localization, interactions, and dynamics within cells, adds another layer of understanding [13]. Information about proteins in organic tissues reflects the results of transcriptional changes and cellular responses. This subfield includes various methods, each with its own limitations and advantages. Combining data from transcriptomics and proteomics offers an additional way to characterize cells and tissues. By integrating these techniques, the changes in translation within a cell can be connected to mechanical forces.

Mass spectrometry (MS) organelle profiling identifies proteins and measures their concentrations in subcellular mixtures. Traditionally, multiple organelles are analyzed together, but this approach is limited due to organelle heterogeneity [13]. More recently, single-organelle profiling has been used to address this issue. This technique enables comparisons between healthy tissues and those affected by diseases, such as cancer. Understanding the effects of viruses such as human cytomegalovirus is improved by comparing protein distributions in healthy and diseased tissues. Combining proteomics profiles of individual organelles within a single cell enhances the statistical significance of results. This method has been shown to capture over 90% of the proteins in a cell, demonstrating high resolution. The primary limitation of this technology is the inability to track proteins over time, as profiling requires lysing the cell. An extension of this method uses multiple single-organelle proteomics profiles to create a protein interaction network within the cell. However, the need for extensive time and high-resolution imaging restricts their practical use. Nonetheless, as new imaging techniques develop, these protein maps will be invaluable for understanding cellular transcription profiles.

Another concept used in spatial proteomics is the application of imaging techniques for subcellular analysis of protein concentrations and locations without cell lysis [13]. Instead of analyzing one subcellular compartment at a time, imaging technologies enable a comprehensive analysis of the cell’s protein profile. Whole-cell imaging has revealed that genetically identical individuals can exhibit different protein profiles due to environmental stressors, such as radiation, force, medication, and others. To image all the proteins of interest, antibodies or fluorescent protein fusions must be introduced without destroying the cell. For antibodies, the field utilizes advancements (Cyclic, sequential, or nucleic acid barcoded methods) from multiparameter immunofluorescence to leverage high-specificity antibodies and multiplexed experiments for thousands of different human proteins. Combining several antibodies with different DNA probes allows the analysis of over 50 proteins in a single imaging session. Despite these advances, creating new antibodies remains a challenging and costly task. An alternative approach involves inserting genes that encode fluorescent proteins. Although technologies like CRISPR-Cas9 have improved the process of creating tags for specific proteins, many proteins are still undetectable by the GFP tags commonly used with this method.

PLA is a unique method for imaging protein interactions *in situ* [14]. These interactions offer insights into how protein chains assemble to enable specific cellular behaviors, such as responding to tensile stress. The proximity probes bring two proteins close enough for the oligonucleotides to be circularized via ligation [15]. Then, RCA amplifies the DNA strands for real-time detection using fluorescence-labeled oligonucleotide hybridization. The technique can also be performed in solution with PCR, rather than in situ, providing an additional way to study cellular protein interactions. This approach confirmed the presence of several proteins involved in stress response at adherens junctions [14]. By combining PLAs with standard immunofluorescence, images show the percentage of one protein interacting with another of interest. Additionally, multiplexed PLAs offer an emerging opportunity for evaluating the clinical performance of drugs [15]. Clinicians can tailor the systems to include patient backgrounds and define various disease-related interactions. As PLAs become more common in spatial research, understanding interactions between proteins and their genetic information will reveal more complex behaviors.

### 2.2. Spatial metabolomics

Spatial metabolomics offers a valuable approach for analyzing chemical properties by imaging to study cellular metabolites. These metabolites are essential to investigate because they represent the final products of numerous biological pathways. Combining metabolite concentration data with spatial analysis emphasizes the importance of these pathways. Because metabolites are challenging to amplify, downstream methods are necessary for analysis [16]. Several techniques are available to measure both the location and amount of metabolites within cells, each offering distinct benefits and challenges.

MSI employs a probe to remove and ionize particles from the cell surface. This rapid process does not destroy the entire cell, enabling multiple analyses on the same sample [16]. The ions are then examined by MS to determine which metabolites are present and their levels. A key challenge with this method is balancing the number of detectable analytes against sensitivity. Another significant issue is that the ionization process can modify the cell before its components are analyzed. Additionally, sample preparation is complex and involves adhering the cells to the coverslip. Focusing on a smaller surface area enables the detection of fewer analytes while improving sensitivity. To address this, researchers are refining existing probes and creating new ones to enhance the measurement of multiple metabolites. Overall, this technology offers a promising approach to repeatedly analyzing metabolites in multiple cells or tissues without destroying them.

DESI employs solvent probes on glass slides to analyze the metabolite profiles of cells, providing an environment similar to *in vivo* conditions [16]. However, DESI cannot distinguish individual cells smaller than 50 *µ*m. SIMS uses an ion beam to image freeze-dried cells or tissues, capable of visualizing interactions between metabolites in single cells smaller than those detectable by other methods. Its drawback is the challenge in identifying more than a few metabolites simultaneously. MALDI involves the use of a laser to analyze cells on substrates, enabling the identification of cell types based on their metabolite profiles. MALDI visualizes more metabolites than SIMS but has lower spatial resolution. Each technique requires different sample preparations and varies in resolution and throughput, factors that should be considered when studying cell metabolites.

SCS involves collecting live cells directly, without prior fixation [16]. Cells or cellular compartments are inserted into nanocapillaries or nanospray emitters, and then exposed to a solvent that lyses them. After lysis, a mass spectrometer examines the metabolites using nano-electrospray ionization. Before analysis, several modifications can be applied to the cellular material to enhance the understanding of drug delivery, metabolites, and other related aspects. Although capable of imaging live cells or compartments with high sensitivity and supporting specific experiments, this method has limitations. The lysing process confines it to 2D cell cultures, which also limits throughput. As a relatively new technology, many labs lack the necessary equipment for full utilization. Instead, they often adapt existing tools, solvents, and protocols to gather relevant data. As SCS grows more common and advances, analyzing metabolites in live cells will be crucial to understanding how cellular pathways influence specific properties.

The single-cell spatially resolved metabolomics (scSpaMet) framework links proteomics and metabolomics data within a tissue [17]. Metal-isotope antibodies improve metabolic imaging through time-of-flight secondary ion MS (TOF-SIMS). These images are integrated with IMC data to enable multiplex protein imaging, identifying cell types with up to 40 markers. This approach also distinguishes various cell types in cancer and detects unique tumor markers. The imaging visualizes metabolite competition among nearby cells and the differentiation of immune cells. Limitations include incomplete coverage of certain metabolites and balancing speed with spatial resolution. Imaging cancer tissues is crucial because of their ability to reprogram metabolism. Monitoring changes in metabolite profiles could enhance cancer diagnosis and therapy. With over 40,000 known metabolites, improving accuracy in identifying cell and cancer types remains challenging [18]. Although MALDI-MSI is common, combining methods from different fields offers a more comprehensive view of cell characteristics. Understanding immune cell interactions advances metabolomics to correlate compounds within immune cells using 3D metabolomics data. Expanding metabolomics to infer cell type properties is essential for elucidating biological pathways.

## 3. Mechanobiology informs multi-faceted cell behavior

Multiple techniques can analyze the combination of signals that create a cell’s specific gene profile. When examining cell behavior, understanding how gene expression changes is crucial. Besides paracrine signaling from other cells, mechanical forces like compression, shear, and stretch are all integrated [19, 20]. Multiple techniques can analyze the combination of signals that create a cell’s specific gene profile. Studying the exact effect of mechanical forces is the goal of mechanobiology. Mechanical forces occur between cells or between the cell and the ECM. Proteins have been identified in cell membranes that act as basic mechano-sensors and can trigger signaling cascades within the cell. Current research aims to connect the mechanical forces experienced by cells to specific changes in gene expression. Additionally, some studies focus on how parts of the cytoskeleton transmit external forces into the nucleus to influence chromatin structure and gene expression, as well as signaling pathways.

Three main types of transcription factors respond to mechanical forces. One type is linked to the cell membrane until phosphorylation in the mechanical sensing pathway causes partial dissociation and eventual nuclear diffusion [3]. Another set in the cytosol relies on signaling pathways and crosstalk to initiate nuclear entry. The third group resides just outside the nucleus and only enters when extracellular stress activates the cytoskeleton. All these transcription factors influence the 3D structure of DNA, including orientation, size, radial distance, and intermingling. Studying how mechanical forces impact gene expression and DNA organization enhances our understanding of cellular signaling in mechanobiology. Techniques to analyze these forces must decompose them into normal and perpendicular components. While many measurements focus on in-plane forces, advanced 3D microscopy now allows tracking forces perpendicular to the imaging plane. This section reviews current methods to monitor cellular forces, highlighting their strengths and weaknesses.

### 3.1. AFM

AFM is a traditional technique used to measure cell mechanics by employing probes to gather data [20]. The resistance encountered by the probe is translated into Young’s modulus for the cell using the Hertz model [20]. AFM is widely used to analyze the surface topography of biological materials and is often combined with other methods for a more detailed understanding of the cell’s biomechanical features. When paired with RT-qPCR, AFM provides a visual map of the cell’s genetic and mechanical characteristics [21]. The expression levels of specific genes have been associated with changes in cancer cell stiffness and their ability to metastasize. Despite its usefulness, AFM has limitations: it can only examine one cell at a time, requires cell fixation, and analyzes cells separately, which may alter their properties had several limitations: it could only monitor one cell at a time, required cell fixation, and processed cells separately, which introduced potential changes in cell properties.

In addition to tracking individual cells, AFM faces other challenges when assessing cellular mechanical properties. Since the probe’s reading is affected by the cell surface, differences in ECM and cell types can alter the outcomes [20]. To mitigate this, AFM is typically applied only to tissues in their natural location. It also struggles to capture tissue heterogeneity unless specialized measurement techniques are employed. Although new methods with various algorithms and approaches aim to enhance this, their effectiveness is still limited.

Force–volume mapping was developed to overcome AFM challenges. This method involves dense raster scans across tissue surfaces to detect variations in Young’s modulus [20]. It requires thick tissue samples, which can be hard to obtain in sensitive areas. Alternatively, small tissue cross-sections can be snap-frozen and thawed, keeping the tissue’s mechanical properties intact while allowing AFM measurements on thinner sections. Although this method addresses some AFM issues, it remains a complex technique that is not yet widely adopted in most labs.

### 3.2. TFM

TFM is another method for mechanical analysis. In a typical TFM procedure, fluorescent beads are distributed among cells under both stressed and unstressed states [4]. The movement of these beads indicates displacement, which can be used to estimate the force on the cell. These calculations assume the substrate is a linear elastic material, with Young’s Modulus remaining constant during force estimation. The silicon substrate is coated with ECM to better replicate *in vivo* conditions. However, tracking the beads and analyzing the data with algorithms is complex and challenging to validate without high-resolution imaging and prior substrate testing, presenting opportunities for improvement.

Modifications to TFM are necessary to precisely track the three-dimensional force profile of cells. Collagen hydrogel is often used to suspend cells and beads, creating an environment similar to the *in vivo* ECM [4]. However, this material behaves as a nonlinear substrate, leading to greater variability in force estimation. Recently, a synthetic, MMP-cleavable PEG hydrogel was developed, which is linearly elastic and facilitates consistent 3D measurements. Nonetheless, the complex calculations required for 3D analysis are not yet straightforward for most laboratories. These limitations in imaging and computation have encouraged the development of more innovative methods for measuring cell forces.

### 3.3. Cells on microfabricated structures

MEMSs are designed to convert cellular forces into electrical signals for recording [4]. They use silicon, known for its reliable electrical properties. When a cell interacts with this environment, it changes the silicon’s electrical output, which researchers then measure. Silicone rubber cantilevers have been used to measure the force output of individual cells. However, a limitation of this technology is that only a few silicone probes can contact a cell at the same time, limiting the ability to analyze forces across space. Additionally, MEMS are complex and difficult to produce, often requiring collaboration with specialized fabrication facilities. Because of these challenges, many researchers have also explored combining traditional probes with mechanical sensing capabilities.

### 3.4. Molecular probing

Ongoing research has led to the development of advanced methods for monitoring cellular forces. One technique uses probes attached to an entropic polymeric molecular spring to measure strain, detecting emission spectra through a fluorophore-quencher pair or a FRET fluorophore pair [4]. These spectra are converted into measurable strain within the molecular spring, allowing for specific readings at individual adhesion sites. However, limitations such as uncertain force calculations have driven scientists to develop DNA hairpin molecules as alternatives. These DNA probes are designed to unfold at specific force thresholds, acting as fluorescent markers at adhesion points. For instance, some DNA probes peel away when subjected to a certain force, embedding into the membrane, which enables microscopy imaging of force distribution along the cell membrane. Though promising, further research is necessary to precisely tune the force required for peeling. Despite their ability to detect forces beyond the peeling threshold, there is currently no established method to translate these measurements into overall cell force estimates.

Another approach involves measuring forces within individual proteins, enabling monitoring inside the cell as proteins deform under specific forces [4]. Although promising, this method faces challenges such as designing suitable protein probes tailored to different cell types. Inserting these probes can damage cells or cause premature protein deformation. Additionally, force measurements based on fluorescent imaging lack clarity, since results from a single molecule cannot easily be scaled to the whole cell. Despite these limitations, these techniques present opportunities to leverage existing fluorescence technology with enhanced mechanical sensing capabilities.

### 3.5. Light-based measurements

Emission and diffraction techniques are emerging methods for force measurement. Using light-based recording, progress is being made to measure more cells simultaneously without sacrificing resolution. MII enables the generation of force readouts for hundreds of cells within a fluid array [22]. By tracking the displacement of micro-reflectors on the cell membrane, it is possible to understand the cell’s natural forces without direct contact, unlike AFM. Although capable of assessing many cells at once, this method does not account for the cell’s native ECM or heterogeneity. Another approach uses Brillouin microscopy, which reduces toxicity compared to traditional force microscopy methods [23]. It minimizes contact by analyzing the emission spectra from photons to estimate mechanical properties, but cannot provide precise force values or detailed force maps, only elastic and viscous properties. This technique can characterize whole tissue samples, but it does not provide the same numerical detail as other methods. Recently, another computational method inferred mechanical signals and patterns in tissues from light-based spatial transcriptomics data [24].

## 4. Cell mechanics applies to several biomedical fields

**Figure 3.**
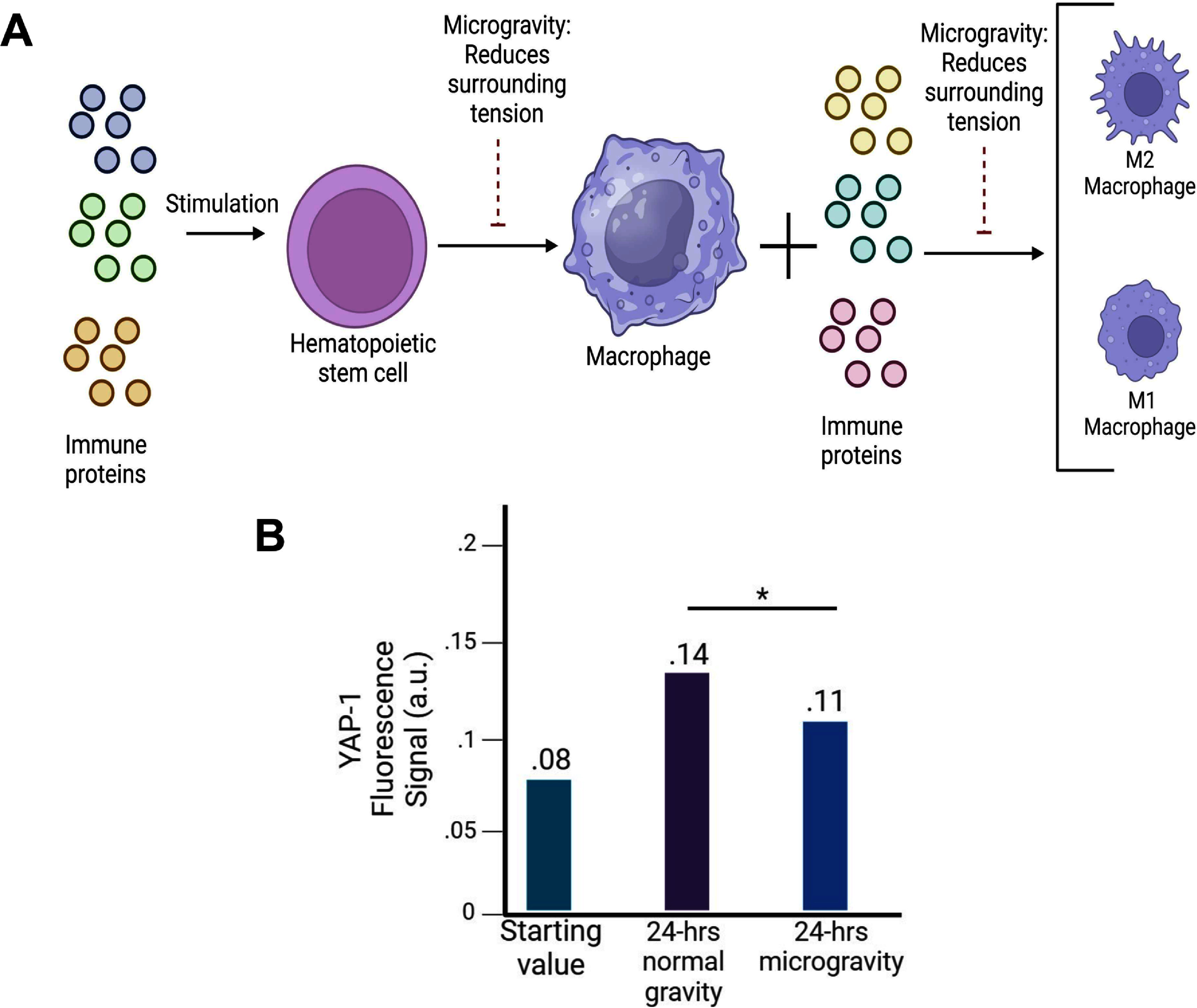
How gravitational forces affect cell differentiation and gene expression. (A) When exposed to microgravity, hematopoietic stem cells and macrophages show reduced differentiation potential. Changes in gravity influence the transcription of specific immune proteins related to differentiation. (B) In brain cancer cells, YAP-1 expression under different gravity conditions indicates that microgravity decreases YAP-1 levels. Created with https://BioRender.com.

Mechanobiology impacts various cell types differently, with gravitational forces influencing gene expression and cell behavior. Applying microgravity modifies how cells express their genes and interact. For example, Yap-1 gene expression decreases in brain tumor cells; this gene acts as a transcription regulator for proliferation genes [25]. Examining Yap-1 levels offers insights into how mechanical forces alter cellular properties (see figure 3). The 3D-printed small assays were sealed except for a syringe inlet hole, incubated with cells, then centrifuged before seeding into the assay. They were incubated at normal gravity for one day, then mounted on a random positioning machine to simulate varying gravitational forces. Most experiments lasted over 24 h, with an average gravity near zero.

Recent study has also explored how immune cells respond to altered gravitational forces. Microgravity suppresses the differentiation of hematopoietic stem cells into macrophages [26]. Five key signaling pathways are downregulated in microgravity, impeding this process (see figure 3). Moreover, microgravity leads to a reduction in dendritic cell production from hematopoietic stem cells, although the reasons for this are still unknown. Neutrophils, vital immune components, are also impacted—some studies report increased neutrophil counts in astronauts post-spaceflight, but the cause remains unclear. Finally, natural killer cells experience heightened apoptosis after prolonged microgravity exposure, with the underlying mechanism still unidentified.

Testing on osteoblast cells during crewless spaceflight missions identified potential reasons for astronaut bone loss [27]. The microgravity experiments involved placing rat osteoblast cells in an uncrewed space shuttle for varying durations. Under microgravity conditions, levels of the vasodilator PGE2 and the insulin-like growth factor IGF-BP-3 increased. Conversely, IGF-BP-4 and IGF-BP-5 showed reduced expression. In summary, alterations in gene expression of growth factors and production of matrix proteins may be key factors in the bone loss astronauts experience in microgravity. Further studies have explored how tissue samples respond to mechanical forces.

### 4.1. Genetic and mechanical links to disease progression

**Figure 4.**
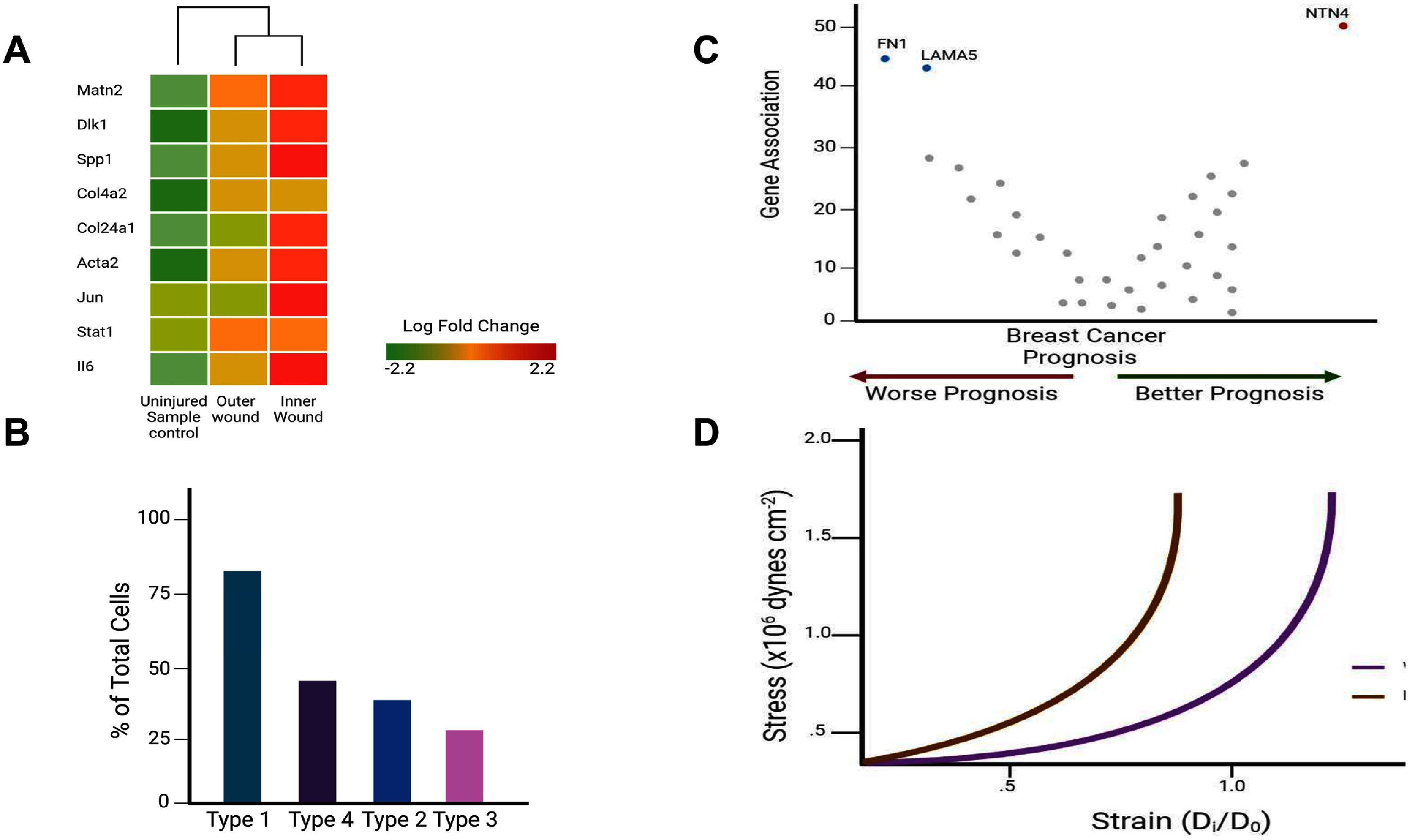
Mechanical forces regulate cell and tissue biology [6]. (A) Heat map showing the changes in gene expression in different areas of a wound. The inner portion of the wound experienced a higher level of expression of critical proliferation genes. (B) Percentage of wound fibroblasts differentiated into four distinguishable transcription profiles (type 1–4). These profiles were linked with distinct gene expression profiles corresponding to certain parts of the wound. (C) Analysis of the expression of specific genes in breast cancer patients and their prognosis. NTN4 positively affected prognosis, while FN1 and LAMA5 produced adverse effects. These genes are related to the stiffness of the basement membrane of epithelial tissue. (D) The comparison of the stress/strain curve for the basement membrane of epithelial tissue in mice when Net4 is present/absent. Removing the Net4 gene resulted in a leftward shift of the stress/strain curve, implying a harder basement membrane. Created in https://BioRender.com.

Mechanobiology also examines tissue structure and gene expression within the body. These tissue properties are linked to important outcomes in diagnosing cancer and other diseases such as atherosclerosis or muscular dystrophy. The relationship between BM stiffness and the risk of cancer cell metastasis has been identified as a potential therapeutic target [6]. Genetic assays on breast cancer patients produced a graphic showing the link between metastasis and the expression levels of various genes. Net4 (NTN4) correlates strongly with increased survival and reduced metastasis, whereas Laminin *α*5 (LAMA5) and Fibronectin (FN1) show the opposite pattern (figure 4). However, only the direct effects of Net4 have been studied.

To study alveolar cancer and melanoma growth, mice with knockout (KO) and wild-type (WT) variants are utilized [6]. Results show a notable increase in cancer metastasis when Net4 is absent. Pressure myography on mesenteric vessels from WT and KO mice revealed that lacking Net4 shifts the stress/strain curve to the left (figure 4). This indicates that Net4 softens the tissue BM, reducing metastasis risk. AFM measured tissue stiffness by inserting a tip through the tissue from the epithelial layer to the endothelial side. This identified a Young’s modulus pattern, pinpointing the BM’s location within the tissue. Findings suggest that Net4 modulates BM stiffness in a concentration-dependent way by altering laminin ternary node complexes. Stiffness drops to about 50% of its original level, while the collagen network likely sustains the remaining structural integrity. Future studies might examine the interaction between collagen networks and tissue mechanics. Identifying genes that influence mechanical changes linked to metastasis could lead to new cancer therapies.

In addition to studying the BM, investigating ECM interactions with various cell types provides another valuable perspective in mechanobiology research. This field covers topics from characterizing tight junctions to exploring cancer growth and muscle repair [28]. Certain genes and receptors, such as integrins and laminins, are particularly relevant to ECM-cell mechanics, influencing cellular behavior at different concentrations. Progress in this area can also be made by designing substrates that mimic *in vivo* conditions, allowing force estimation techniques and experiments to connect ECM changes with gene expression shifts. A key focus is the growth of microtissues in response to mechanical forces from the ECM, as seen in studies where increased contractile forces in myofibroblasts facilitate wound closure [29]. Tissue damage alters cellular mechanical profiles, triggering genetic and behavioral responses that lead to further mechanical changes. This feedback loop in mechanobiology is essential not only for wound healing but also for many other processes involving cellular phenotypic shifts.

Finally, spatial transcriptomics has relevant applications in cancer research. Understanding how normal and malignant cells react to mechanical forces provides a new angle for developing future treatments. By analyzing cancer cells’ mechanical and transcriptional characteristics, treatments and diagnostic tools can become more accurate in detecting and monitoring diseased tissues. A high-throughput technique to study the mechanotransduction in cancer and immune cells helps clarify their responses and behaviors [30]. Changes in cell viability can be tracked by altering the FSS applied to various cells in combination with therapeutics. This pathway paves the way for future research to examine the effects of FSS on cancer without relying on *in vivo* experiments. The findings support the integration of mechanical forces with conventional therapies to achieve better patient outcomes and a more comprehensive understanding of mechanotransduction in certain cancers.

### 4.2. Connecting mechanobiology to wound healing

Mechanobiology influences tissue injury repair beyond tissues themselves. Wound healing involves a dynamic interaction between the ECM and nearby cells. The ‘Rainbow Mouse’ system was used to trace the lineage of fibroblasts near a wound [29]. This system employs four-color reporters at the Rosa26 locus, making progeny carry the same ID as their parent. Fibroblasts close to a wound clone more frequently than in untouched areas. Data shows that gene expression linked to increased cloning varies among fibroblasts, with the inner wound area displaying different gene expressions than the outer part (figure 4). Analyzing gene expression changes identifies four fibroblast types in wound healing: ‘mechanofibrotic’ (ArchR-cluster 1), ‘activated-responder’ (ArchR-cluster 2), ‘remodeling’ (ArchR-cluster 3), and ‘proliferator’ (ArchR-cluster 4). Understanding fibroblast behavior is enhanced by studying how wound healing is affected when mechanotransduction pathways are blocked with FAK inhibition. Wounds exhibit less scarring and resemble unwounded skin more closely when ArchR-cluster 1 is most suppressed.

**Figure 5.**
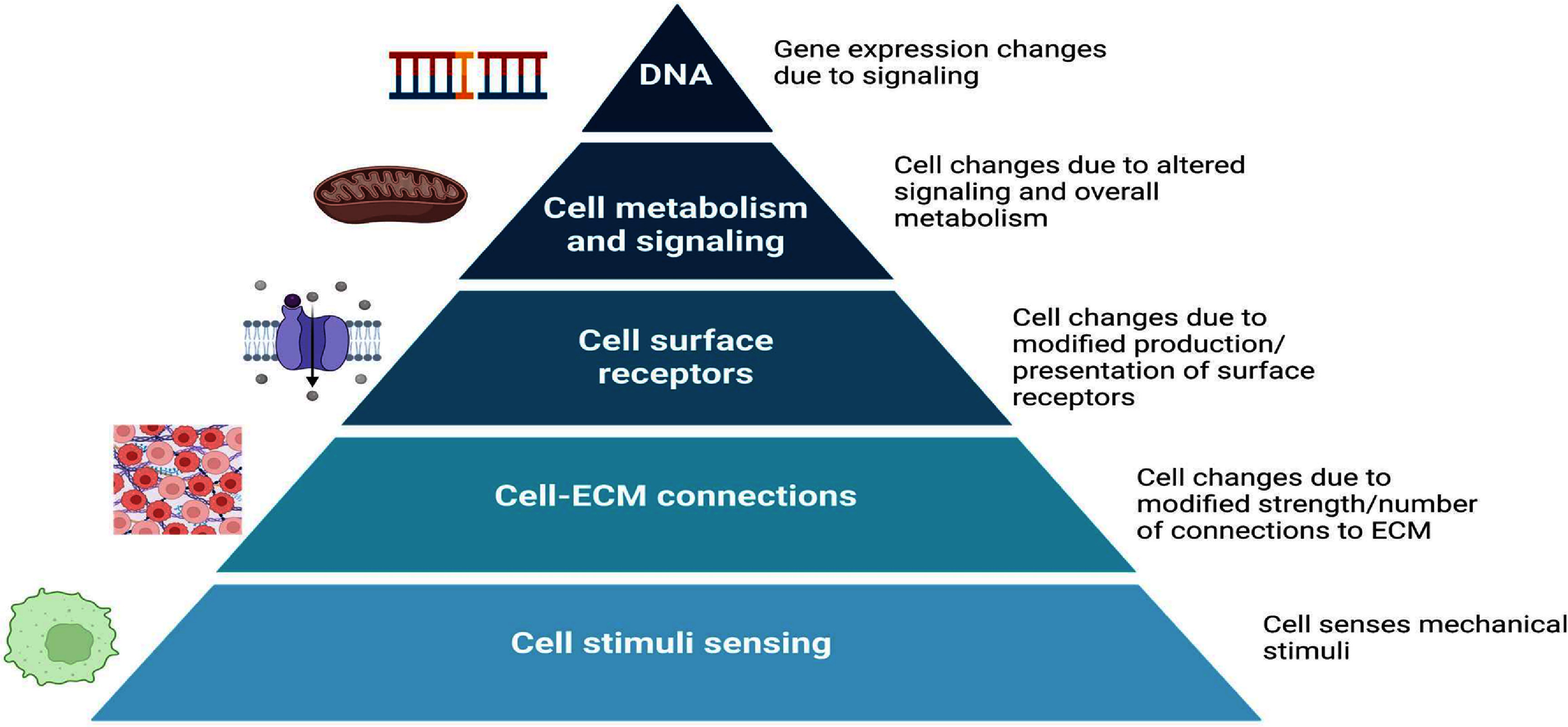
Hierarchy of cell regulation in mechanobiology. All cells sense stimuli at several locations. In mechanobiology, cell responses can be measured via their mechanical behavior. Cell-ECM connections can also be measured in response to changes in mechanical forces on the cell. When the changes are cell-specific, changes in cell surface receptors, cell metabolism, and DNA expression can all be monitored due to changes in mechanical forces. Created in https://BioRender.com.

**Figure 6.**
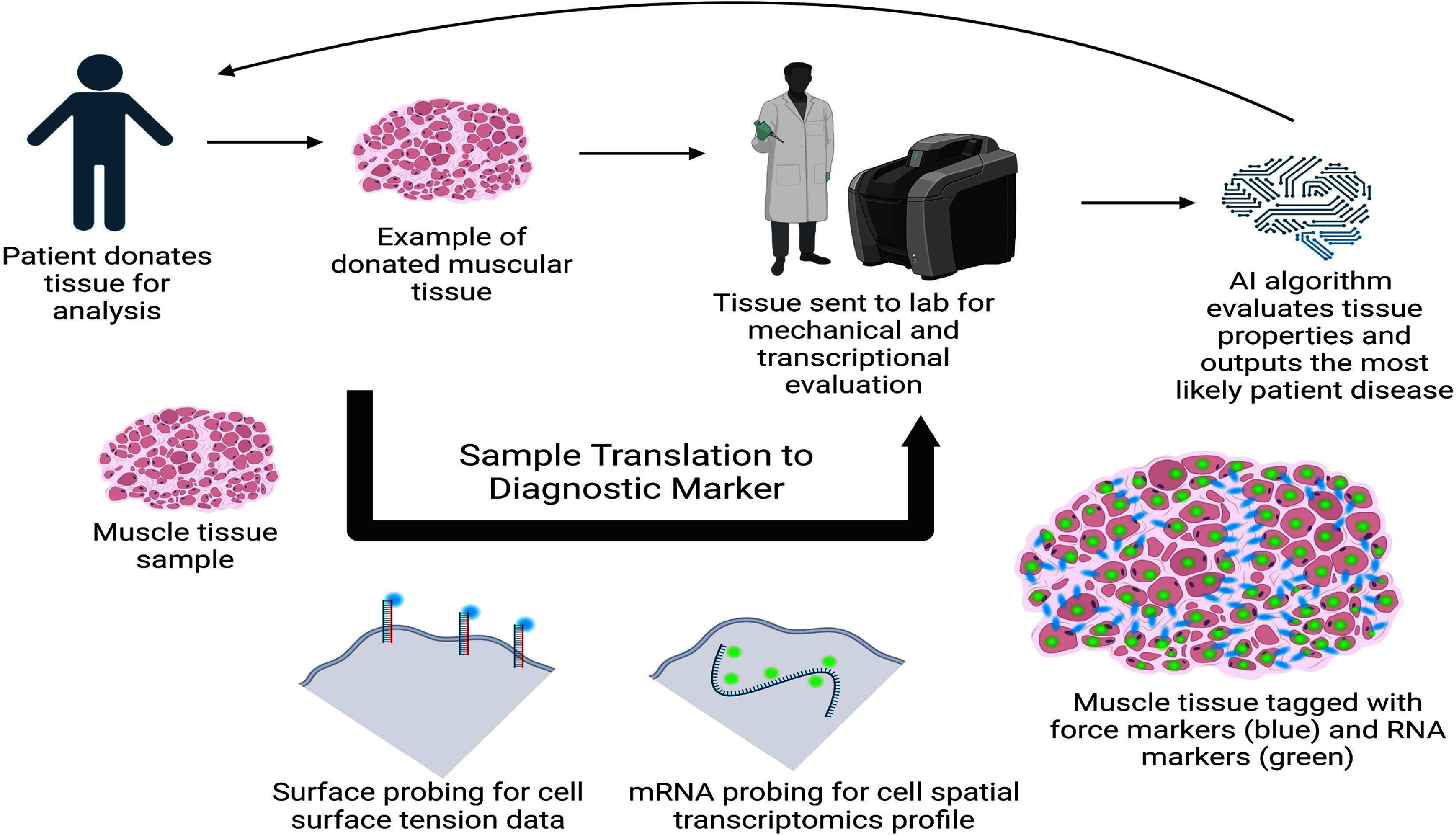
Translation of spatial mechanogenomics to diagnosis. Several AI algorithms are currently being developed and used for diagnostic purposes. These algorithms primarily focus on imaging analysis, a pathway that can be applied to spatial transcriptomic and mechanobiological data. The patient would donate tissue for study, which the pathologist would tag with mechanical and transcription markers to analyze via fluorescence microscopy and produce the image on the bottom right. The comparison of healthy and non-healthy images would train the AI algorithm to output the condition the patient is suffering from. The pathway could be applied to several tissue types, but will likely focus on epithelial, muscle, and blood tissue/cells since they can be more easily sampled. Created in https://BioRender.com.

In summary, many applications arise from understanding cell mechanobiology and transcriptomics. Discovering how environmental forces regulate certain genes can drive research into the feasibility of long-term space missions. Analyzing gene regulation during wound healing can lead to therapies that minimize scar tissue formation after injury. Understanding cellular responses to neighboring cells is crucial for designing effective transplants or grafts. Integrating biological data across various physical and temporal scales can improve patient diagnosis (see figure 5). A deeper understanding of cell transcription fosters stronger links between mechanical forces and the genes that control cell behavior or stem cell fate. Mapping transcription profiles of individual cells enables more accurate identification of tissue boundaries, allowing for precise incisions and targeted treatments. A mechanobiological workflow combining gene, protein, and mechanical assays provides valuable information to pathologists and assistive algorithms about a patient’s health (see figure 6).

### 4.3. Increasing the role of cell mechanics in diagnostics

Beyond therapeutics and disease assessment, mechanobiology can enhance diagnostics as well. Direct cell force measurements relate to the mechanotransduction pathway, which, when disrupted, can signal diseases such as and heart conditions [31]. Mechanical responses of tissues, especially chemical and electrical changes, can help diagnose illnesses. When cells encounter altered mechanical forces, subtle chemical and electrical shifts can be precisely detected and analyzed with machine learning to evaluate tissue stiffness and related diseases like cardiac hypertrophy [31]. These measures of force, both direct and indirect, are linked to various diseases outlined in this paper (table 1). Moreover, real-time assessments of conditions like stenosis and osteoarthritis are possible using these direct measurements [32]. Devices that monitor stresses in bloodstreams and airways serve as secondary diagnostic tools and are likely to improve with AI integration [31, 33]. Overall, applying mechanobiology to study disease development and wound healing, as well as its use in diagnostic tools, shows its promising future in biomedical research.

## 5. Integration of spatial omics and mechanobiology

The integration of spatial omics and mechanobiology deepens our understanding of cell communication via chemical and mechanical signals. Recent efforts emphasize particular aspects of this developing field. Different cell types under various mechanical stresses display distinct profiles that warrant future analysis. Moreover, there is an increasing focus on exploring how a cell’s transcription, proteomic, and mechanical profiles are interconnected. Studying these relationships can enhance patient outcomes and enable the development of advanced cellular simulations.

To accurately simulate a cell’s properties, reliable data is essential for developing a consistent model for spatial mechano-transcriptomics. This model needs information about cell boundaries and their neighboring cells. A mechanically sensitive model is created using fluorescent images of cell contours in tissues. These images supplied spatial data about the cells and their neighbors, exerting pressure on them. Precise image-based segmentation is employed to characterize the cells’ spatial environment and junctions. After building the model, certain assumptions simplified calculations. The main limitation is excluding cells with four-fold vertices to ensure all junctions are convex, as these are shared among four or more cells. Convex junctions are crucial for the VMSI mechanical inference approach [24], which employs non-planar triangulation of junction tensions to model cellular pressure and tension. This method confirms that tension between cells is higher at boundaries, facilitating tissue formation and maintenance. The tension is modeled by incorporating ephrin ligand-receptor signaling, which enhances actomyosin contractility at junctions. Several genes affected by mechanical forces are identified through correlation with spatial transcriptomic imaging. However, the process heavily relies on segmentation quality, which cannot perfectly separate cells in all tissue types. Additionally, since the imaging technique is limited to 2D, the scope of findings is restricted. Despite these challenges, this technology offers insight into a future where simulated cells could become valuable testing tools. These methods can be summarized to provide an overview of mechanobiology (table 3).

**Table 3.** Summary of the main features of many mechanobiology testing methods.

	Force-based microscopy	Physical probes	Computer simulation	Light-based microscopy
Methods	AFM, TFM	DNA Hairpin, Protein-specific, MEMS, Expansion microscopy	Junction simulation	Brillouin microscopy, MII
Modality	Cantilever or fluorescent bead displacement	Force-sensitive probe activation	Cell-cell junction imaging	Photon emission spectra, reflector displacement
Metric	Cell adhesion forces, displacement force (2–120 nN)	Force activation of fluorescent markers	Simulation of cell-cell forces at junctions	Displacement of photons/micro-reflectors to calculator
Resolution	Sub-cellular (2 *µ*m)	Sub-cellular (0.2 *μ*m)	Single-cell	Sub-cellular (1.5 *μ*m)
Sample area	Up to 1000 cells	Can scale up with additional probes, typically limited to 600 cells for time	Can scale with processing power and detail of image used for simulation	Can scale up with larger equipment
Availability	Several AFM machines, several confocal microscope options	Several MEMS machines, a limited selection of protein/DNA probes	Standard immunoblotting and spatial transcriptomics images for reference, minimal availability of simulation method	Both are custom modifications of existing technology
Required instruments	AFM device, custom cantilevers, fluorescent beads, confocal microscope	MEMS sensor, fluorescent microscope for probe readings	Fluorescence microscope,mass spectrometry imaging, specific simulation algorithm for junction forces	Brillouin microscope, interference microscope, several modifications to both devices
Reference	[4, 20, 21]	[4, 34]	[24]	[22, 23]

Another emerging technique involves using fluorescent markers that activate only when subjected to tensile force. As previously noted, these force-sensitive molecular probes are a promising avenue for exploring how cells interact with their environment. When cells are carefully seeded onto specialized well-plates, experiments can visualize the cell boundary and measure the forces exerted on the surroundings [34]. Providing both spatial and mechanical information enables further investigation into how cell forces concentrate in specific regions over short time frames. Creating time-lapse videos of cell movement and activation of these tension probes helps deepen the understanding of cell motility [35]. These methods can be applied to various cell lines and tissues to gain insights into healthy and diseased states in the human body. Additionally, these videos and mechanical data can inform new mechanically driven models to simulate cell surface properties and behavior within their environment.

## 6. Conclusion

Mechanobiology examines tissues’ physical interactions and responses, but faces challenges in spatially resolving cellular details. This report showcases examples of how mechanobiology is used to better understand how mechanical forces impact biology at the cellular level, including applications like wound healing and space exploration. Spatial transcriptomics is a rapidly expanding field with many promising avenues for visualizing cellular mechanical features, though it often neglects tissue mechanics. While different methods are available to estimate tissue properties, more progress is needed to directly visualize mechanical data. Learning from spatial proteomics and metabolomics, spatial transcriptomics could produce visualizations of various cell behaviors, including mechanical aspects. Overall, integrating mechanobiology with spatial transcriptomics can advance biomedical engineering and support more accurate treatments by combining physical and genetic cellular information. These developments can also enhance insights into cell communication, tissue structure, and diseases such as cancer. Although the influence of physical forces on biology is not new, the detailed visualizations provided by spatial omics will improve quantification and encourage more informed collaboration among healthcare professionals and researchers.

## Data Availability

No new data were created or analysed in this study.

## Data access

No raw data was used in this perspective.
